# Stabilization and Preservation of Bioactive Compounds in Black Elderberry By-Product Extracts Using Maltodextrin and Gum Arabic via Spray Drying

**DOI:** 10.3390/foods14050723

**Published:** 2025-02-20

**Authors:** Zorana Mutavski, Senka Vidović, Zorica Lazarević, Rita Ambrus, Anett Motzwickler-Németh, Krunoslav Aladić, Nataša Nastić

**Affiliations:** 1 Faculty of Technology, University of Novi Sad, Boulevard cara Lazara 1, 21000 Novi Sad, Serbia; zmutavski@mocbilja.rs (Z.M.); senka.vidovic@uns.ac.rs (S.V.); 2 Institute for Medicinal Plants Research “Dr. Josif Pančić”, Tadeuša Košćuška 1, 11000 Belgrade, Serbia; zdrinic@mocbilja.rs; 3 Faculty of Pharmacy, Institute of Pharmaceutical Technology and Regulatory Affairs, University of Szeged, Eötvös Street 6, H-6720 Szeged, Hungary; ambrus.rita@szte.hu (R.A.); nemeth.anett@szte.hu (A.M.-N.); 4 Faculty of Food Technology Osijek, University of Josip Juraj Strossmayer in Osijek, Franje Kuhača 8, 31000 Osijek, Croatia; krunoslav.aladic@ptfos.hr

**Keywords:** elderberry pomace, spray drying, ultrasound-assisted extraction, preservation, food waste utilization, powder formulation, sustainable development

## Abstract

This study investigates the encapsulation efficiency and physicochemical properties of black elderberry pomace powders obtained by a spray-drying process employing maltodextrin and gum arabic as encapsulating agents. The formulations SD 1 to SD 6 were prepared in different ratios, from 100% maltodextrin (SD 1) to 100% gum arabic (SD 6). The encapsulation yield (EY) ranged from 75.36% to 83.84%, with SD 1 achieving the highest EY of 83.84%. Particle size analysis revealed average sizes between 1.73 μm and 2.20 μm, with SD 2 showing a uniform distribution. Flow and compressibility studies showed that SD 4 (40% maltodextrin, 60% gum arabic) had better flow properties (Carr index of 27.34) compared to SD 1 (Carr index of 39.91). The retention of bioactive compounds showed that SD 1 retained cyanidin 3-O-sambubioside at 17.55 mg/g and cyanidin 3-O-glucoside at 14.20 mg/g, while SD 4 showed high efficiency for kaempferol derivate 1 (97.86% in SD 5) and kaempferol derivate 2 (98.57% in SD 4). Overall, SD 4 proved to be the optimal formulation, significantly enhancing the stability and bioavailability of elderberry extract in food and nutraceutical applications. This is attributed to its high encapsulation efficiency and effective retention of bioactive compounds, making it an ideal candidate for incorporation into functional foods and dietary supplements aimed at promoting health benefits.

## 1. Introduction

Black elderberry (*Sambucus nigra* L.) is widely known for its rich content of bioactive compounds, particularly polyphenols, flavonoids, and anthocyanins, which contribute to its numerous health-promoting properties [1,2,3]. These compounds have been extensively studied for their antioxidant [4,5], anti-inflammatory [6], and antiviral properties [7,8], making black elderberry a valuable ingredient in dietary supplements, functional foods, and pharmaceuticals. Polyphenols’ elevated antioxidant capacity helps to neutralize free radicals, decrease oxidative stress, and potentially reduce the risk of chronic diseases, while the flavonoids and anthocyanins contained in elderberries are known to support immune function and provide natural protection against viral infections [8]. Despite these recognized benefits, an important by-product of elderberry juice production, known as pomace, remains underutilized. This pomace, which consists of the peel, seeds, and pulp left behind after juice production, is a concentrated source of the same beneficial bioactive compounds found in juice [9]. However, instead of being fully utilized, elderberry pomace is often discarded or only used to a limited extent. This is a missed opportunity to extract valuable compounds that could be further utilized in dietary supplements or other health-related products. Maximizing the use of elderberry pomace is not only in line with sustainable practices by reducing the amount of waste but also provides an opportunity to harness the untapped bioactive potential and thus increase the value of this underutilized by-product [10,11]. In terms of sustainability, the use of elderberry pomace is in line with several Sustainable Development Goals (SDGs), in particular Goal 12, which focuses on responsible production and consumption. Specifically, target 12.3 seeks to reduce global per capita food waste at the retail and consumer levels and minimize food losses throughout production and supply chains [12].

Efficient extraction of valuable ingredients from black elderberry pomace can be achieved with ultrasonic-assisted extraction (UAE), an environmentally friendly extraction technique that increases the yield and quality of extracts [13]. UAE employs ultrasonic waves to break open the cell walls of the plants, resulting in shorter extraction time, lower solvent consumption, and improved recovery of heat-sensitive bioactive compounds [13,14,15]. Despite these advantages, the stability of UAE extracts poses a major challenge, particularly due to the susceptibility of compounds such as anthocyanins to degradation under environmental factors such as light, heat, and oxygen [16]. To deal with these stability issues, encapsulation techniques such as spray drying have been developed to protect bioactive compounds during storage and processing.

The pharmaceutical and food industries are constantly striving to improve drug formulations and nutraceuticals to enhance therapeutic efficacy, patient compliance, and overall treatment outcomes [17]. Among the various dosage forms, solid preparations, including tablets, powders, capsules, and granules, have gained considerable popularity due to their numerous advantages over liquid formulations. These advantages include greater chemical and physical stability, ease of storage and handling, precise dosing, and longer shelf life [18]. Solid dosage forms are particularly beneficial for drugs and nutraceuticals that are prone to hydrolysis or oxidation, as they are less susceptible to degradation and can maintain their efficacy over time [19,20].

In addition to their inherent stability, solid dosage forms offer greater convenience during storage, handling, and transportation. This makes them ideal for a wide range of pharmaceutical and nutraceutical uses. One of the most versatile and efficient encapsulation techniques is spray drying, which converts liquid extracts into dry particles with precise size and high encapsulation efficiency [21,22]. Spray drying is compatible with a variety of hydrophilic and hydrophobic compounds, enabling customizable product properties tailored to specific applications [22,23]. In addition, the process is suitable for large-scale manufacture due to its scalability and cost-effectiveness, which expands its applicability in various industries, and it can be applied to underutilized by-products. Spray drying is chosen over particles from gas-saturated solutions (PGSSs) and lyophilization due to its faster processing time, lower operational costs, and ability to produce customizable powders at scale, making it more suitable for industrial applications in sectors like food and pharmaceuticals [24].

Spray drying is widely used as it converts liquid extracts into stable powdered forms while preserving the bioactivity of the encapsulated compounds and is reported to account for around 40% of the global drying market. Common applications include the production of powdered milk, instant coffee, and various fruit powders that contribute to food convenience [25]. Maltodextrin and gum arabic are commonly used as carriers for spray drying due to their excellent encapsulation properties and compatibility with numerous bioactive compounds [26,27]. Maltodextrin, a polysaccharide derived from starch, offers high solubility, excellent film-forming ability, and cost-effectiveness and forms an amorphous matrix that protects and stabilizes the encapsulated compounds [28]. Gum arabic, a natural secretion from acacia trees, is known for its emulsifying and stabilizing properties and facilitates the formation of microcapsules that increase encapsulation efficiency and enable controlled release [29].

The primary goal of this study is to optimize the drying process for UAE extracts from underexplored black elderberry pomace to enhance the stability and preservation of its bioactive compounds. By systematically varying the process parameters and the ratio of encapsulating agents, this research aims to identify the optimal conditions for producing a stable, high-quality powdered extract. The novel findings from this study are predicted to contribute to the development of more effective natural product-based ingredients for use in various health-related applications, ultimately reducing waste and adding value to black elderberry pomace.

## 2. Materials and Methods

### 2.1. Herbal Material, Extract Preparation, and Chemicals

Black elderberry pomace (*Sambucus nigra* L.) was sourced from NISHA d.o.o., a company in Belgrade (Serbia) specializing in forest fruit harvesting and processing. The raw material underwent drying in a vacuum oven (Kemoservis-Fotomaterial, Ljubljana, Slovenia) and was ground using a mixer (Bosch, Gerlingen, Germany). The extraction parameters for phenolic compounds were based on our previous studies [30], using a 30% diluted EtOH solvent and a 7 min ultrasound treatment at an amplitude of 60% with an ultrasound probe (Hielscher Ultrasonic GmbH, Stuttgart, Germany). The extraction ratio was kept constant at 1:10 (pomace-to-solvent). After extraction, the prepared extract was further analyzed. Extraction yield was 29.04%. Consequently, nearly one-third of the initial drie waste material was utilized.

HPLC grade acetonitrile (Merck, Darmstadt, Germany) and ultrapure water from a Milli-Q system (Merch Millipore, Guyancourt, France) were used in the analysis. The phenol standards with a purity over 96% were purchased from Extrasynthese (Genay, France). Maltodextrin (dextrose equivalent DE 16.5) and gum arabic (Sigma-Aldrich Chemie GmbH, Steinheim, Germany) were employed as a carrier material. All other chemicals in the study were of analytical grade and did not require further purification.

### 2.2. Encapsulation Process—Spray Drying

The prepared polymer solutions were dried using a Büchi Mini Spray Dryer B-290 laboratory device (Flawil, Switzerland). Experimental conditions were set as follows: volume of extract 100 mL, feed flow solution 1.2 mL/min, inlet temperature 120 ± 1 °C, outlet temperature 60 ± 2 °C, nozzle diameter tip 150 μm, atomization air flow 831 L/h, and aspiration 100%. Maltodextrin and gum arabic were used as a carrier material (Table 1) in a 1:2 (carrier:dry mass of extract) ratio. The obtained powder was collected in closed glass bottles and protected from air and humidity.

### 2.3. Physical and Chemical Characterization of Powders

#### 2.3.1. Encapsulation Yield (EY) and Moisture Content (MC)

The EY (Equation (1)) was calculated by comparing the actual dry powder mass (m_p_) collected in the receiving vessel of the device and the expected powder mass (consisting of the mass of the dry residue of the collected extract and the carrier mass, m_ep_):

EY (%) = m_p_/m_ep_ × 100, (1)

The MC of the prepared powders was determined thermogravimetrically by heating the sample at 105 °C until a constant mass was achieved, using a halogen moisture analyzer (HB43-S, Mettler Toledo, Columbus, OH, USA). The moisture content was calculated using Equation (2) (mass of moisture, m_m_; mass of sample, m_s_):

MC (%) = m_m_/m_s_ × 100, (2)

#### 2.3.2. Bulk and Tapped Densities

To determine the densities, samples were placed into 5 mL graduated cylinders, and bulk density (ρ_bulk_) was calculated from the mass-to-volume ratio. Tapped density (ρ_tap_) was determined using the STAV 2003 Stampf volumeter (Engelsmann A.G., Ludwigshafen, Germany), with results obtained from the mass-to-tapped volume ratio. The procedure for measuring bulk and tapped densities of powders is outlined in Chapter 2.9.34 of the European Pharmacopeia. Flowability and cohesion were assessed by calculating the Carr index (CI) and Hausner ratio (HR) using Equations (3) and (4):

CI = (ρ_tap_ − ρ_bulk_)/ρ_tap_ × 100, (3)

HR = ρ_tap_/ρ_bulk_, (4)

#### 2.3.3. Particle Size and SEM Analysis

The morphology, particle size, and particle diameter distribution of the obtained powders were analyzed using scanning electron microscopy (SEM). A Hitachi S4700 (Hitachi Scientific Ltd., Tokyo, Japan) was used to capture the SEM pictures. Before imaging, the powdered samples were sputter coated with gold in argon atmosphere to enhance their conductivity. High-resolution micrographs were obtained by employing the following parameters: 10 kV high voltage, 10 mA amperage, and 1.3–13.1 mPa air pressure. The SEM pictures of microparticles were examined and evaluated using ImageJ Software 1.53t.

#### 2.3.4. HPLC Analysis

An Agilent 1200 RR system (Agilent, Waldbronn, Germany) with a diode array detector was employed for the analysis of phenolic compounds. The separation was performed on a reversed-phase Lichrospher RP-18 column (250 mm × 4 mm, 5 μm, Agilent, Waldbronn, Germany), with the column temperature set at 25 °C. The mobile phase consisted of solvent A (10%, *v/v* solution of formic acid in water) and solvent B (acetonitrile) using the following gradient elution profile: 1% B 0–0.5 min; 1–7% B 0.5–1 min; 7% B 1–4 min; 7–10% B 4–7.5 min; 10–14% B 7.5–11.5 min; 14–25% B 11.5–15.5 min; 25–40% B 15.5–18.5 min; 40–75% B 18.5–22 min; 75% B 22–25 min. A 10 μL sample volume was injected, with a flow rate of 1 mL/min. Detection occurred at wavelengths of 290, 350, and 520 nm. Rutin content was quantified based on a calibration curve, with results reported in milligrams per gram of dry weight (mg/g powder). The calibration curve exhibited a correlation coefficient of 0.9999, demonstrating good linearity in the range of 50 to 500 µg/mL.

The encapsulation efficiency (EE) was calculated as the ratio of quantified to theoretically expected bioactive compound content in the powders (%). The expected compound concentration corresponded to the theoretical amount present, accounting for dilution due to carrier addition at an extract-to-carrier ratio of 1:6.

### 2.4. Statistical Analysis

The analyses were conducted in triplicate, and results were expressed as means ± standard deviations (SDs). Differences between means were considered significant at a confidence level of *p* < 0.05. Statistical analysis was performed using one-way ANOVA, followed by Tukey’s test for multiple comparisons.

## 3. Results and Discussion

### 3.1. Powder Characterization

#### 3.1.1. Encapsulation Yields and Moisture Contents of Encapsulated Extracts

In the present study EYs are relatively high across all samples, ranging from 75.36% to 83.84% (Table 2), with no significant statistical differences observed (*p* > 0.05), indicating that both maltodextrin and gum arabic are effective encapsulating agents under the given spray-drying conditions. However, a trend is observed where the highest EY is obtained with 100% maltodextrin (SD 1), and the yield tends to slightly decrease as the proportion of gum arabic increases, reaching a lower EY in SD 4 (40% MD, 60% GA). Interestingly, the yields increase again for SD 5 and SD 6, where gum arabic is the dominant or sole encapsulating agent.

Maltodextrin and gum arabic are both commonly used encapsulating agents in spray drying, with each having distinct properties that influence encapsulation efficiency [27]. Maltodextrin is known for its good solubility and ability to form a protective matrix [31], while gum arabic offers excellent emulsification properties and film-forming ability [32]. The literature suggests that maltodextrin typically results in higher encapsulation yields due to its ability to rapidly form a matrix around the core material, minimizing the loss of volatile or sensitive compounds through the spray-drying process [26]. The results for SD 1, where 100% maltodextrin was used, are consistent with this, showing the highest EY. Gum arabic, while providing strong film-forming properties, may lead to slightly lower encapsulation yields when used alone or in higher proportions, as seen in SD 4. However, it still achieves relatively high yields, as evidenced by the results for SD 6 (81.25 ± 1.12%), which aligns with literature reports suggesting that gum arabic can effectively encapsulate certain types of compounds, particularly when combined with other wall materials [33]. The mixture of maltodextrin and gum arabic, as seen in SD 2 to SD 5, allows for a balance between the properties of both encapsulating agents, leading to consistently high yields. This suggests that the mixture of maltodextrin and gum arabic could be optimized depending on the specific requirements of the encapsulated material, such as solubility, stability, and release profile.

The MC of the powders shows a clear trend where SD 1, with the highest moisture content (5.63 ± 0.15%), stands out as the least stable in terms of flowability and compaction, which could negatively impact its handling properties. SD 2, with a slightly lower moisture content (4.95 ± 0.20%), still retains relatively good moisture but shows improved flow characteristics compared to SD 1.

Samples SD 3 through SD 6, with MC below 4.20%, exhibit better overall stability. SD 4, SD 5, and SD 6, in particular, demonstrate significantly lower MC (3.85 ± 0.10% to 4.10 ± 0.09%), aligning with their superior flowability and compaction properties. These lower moisture levels contribute to enhanced powder stability, making them more suitable for processing and storage. The reduced MC in these samples suggests they are less prone to clumping and may offer extended shelf life.

The MC of the obtained powders could be influenced by multiple factors, including inlet and outlet temperatures, drying duration, feed flow rate, and the physicochemical properties of the carrier material. While maltodextrin exhibits lower hygroscopicity than gum arabic [34], powders with a higher proportion of maltodextrin paradoxically retain more moisture, indicating that MC was governed by a complex interplay of factors rather than a single parameter. Furthermore, despite maintaining consistent drying conditions across all samples (except for variations in carrier composition), minor inherent process fluctuations can contribute to differences in the final MC. One of the limiting factors in this regard could be the elevated hygroscopicity of gum arabic, which can lead to higher moisture retention in powders with higher proportions of this carrier.

#### 3.1.2. Flowability and Compressibility Parameters of Powders

Spray-dried powders are usually evaluated according to their flowability and compressibility, which are decisive for their performance in various applications. Parameters such as bulk density (BD), tapped density (TD), Carr index (CI), and Hausner ratio (HR) provide information on the handling and processing properties of these powders [35,36]. In this study, the flowability and compressibility of elderberry extract powders labeled SD 1 to SD 6 were investigated to determine their suitability for different applications. The results highlight the differences in the behavior of the powder and provide valuable information for optimizing the spray-drying conditions and improving the handling of the powder.

The BD of the powders ranged from 75.36 ± 3.54 g/L for SD 4 to 83.84 ± 3.39 g/L for SD 1, with no significant statistical differences between the samples (*p* > 0.05). This consistency in BD suggests a similar packing behavior among the powders when loosely filled, indicating uniform particle distribution and size across the samples. TD values exhibited greater variability, ranging from 247.22 ± 3.02 g/L for SD 4 to 277.14 ± 3.53 g/L for SD 3. SD 3, with the highest TD, suggests a more cohesive powder that compacts more effectively under mechanical tapping.

The CI, a measure of powder compressibility, showed marked differences among the samples. SD 1 had the highest CI at 39.91 ± 0.76, indicating the poorest flowability, while SD 4, SD 5, and SD 6 displayed significantly lower CI, around 27, reflecting much better flow properties (*p* < 0.05). The lower CIs in these samples suggest less compressibility and a reduced tendency to form aggregates. HR values followed the trend observed with the CI. SD 1 exhibited the highest HR at 1.66 ± 0.08, consistent with poor flowability. In contrast, SD 4, SD 5, and SD 6 had significantly lower Hausner ratios (1.37–1.38), indicating superior flow characteristics (*p* < 0.05). The statistically significant differences between SD 1 and the other samples highlight its less favorable flow properties.

The analysis reveals that SD 1 is characterized by the poorest flowability and highest compressibility among the samples, as evidenced by its higher CI and HR. This suggests that SD 1 may pose challenges in processing and handling due to its propensity to form aggregates or its reduced free-flowing nature. In contrast, SD 4, SD 5, and SD 6 demonstrate superior flowability and compressibility, making them more suitable for applications where consistent powder handling is critical. The combination of maltodextrin and gum arabic improves particle properties by balancing the rigid, moisture-resistant matrix of maltodextrin with the flexible, moisture-retentive, and emulsifying characteristics of gum arabic, enhancing stability, flowability, and encapsulation efficiency [37]. Gum arabic’s ability to form a protective matrix around bioactive compounds further contributes to improved retention by reducing moisture uptake and preventing degradation. Its emulsifying properties also aid in the uniform distribution of bioactives, ensuring efficient encapsulation and controlled release. These properties suggest that the formulation could be successfully scaled up for industrial applications. The stability and encapsulation efficiency, alongside the favorable flowability of the powders, indicate that a scale-up process could be feasible for large-scale production. These conclusions can guide further optimization of formulation and processing conditions to enhance the flow properties of powders, particularly for those exhibiting higher CI and HR. These findings provide insight into the optimization of spray-drying processes or the selection of additives that could improve the flowability of the powders, ensuring better performance in their intended applications.

#### 3.1.3. Morphological and Particle Size Parameters

**Figure 1:**
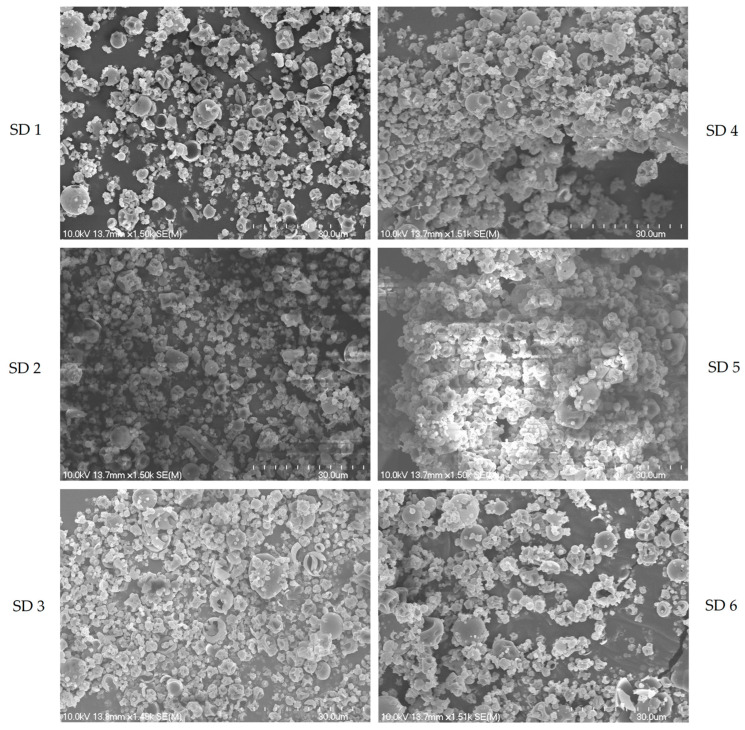
SEM analysis of black elderberry pomace extract microparticles (1500× magnification, 30 µm scale).

The SEM analysis of the powders SD 1 to SD 6 shows a close correlation with their particle size distribution data, highlighting the differences between samples (Figure 1). SD 1, which has the smallest particle size (1.73 ± 1.27 μm), exhibits relatively fine particles in the micrographs. These particles appear more variable in size, consistent with the high standard deviation observed in their particle size distribution, suggesting a less uniform formation during spray drying.

In comparison, SD 2, with a particle size of 1.94 ± 0.08 μm, shows a more uniform particle distribution, as indicated by the lower standard deviation. The SEM images of SD 2 likely reflect more consistently shaped and sized particles with smooth surfaces, contributing to better flow properties and compaction behavior, as observed in its bulk and tapped density measurements.

Similarly, for powders SD 3 to SD 6, with particle sizes ranging from 2.05 ± 0.10 μm to 2.20 ± 0.58 μm, the SEM micrographs show slightly larger particles, with SD 4 exhibiting the largest particle size (2.20 ± 0.58 μm). The smooth and spherical morphology of these powders remains advantageous for stability and handling, though the slight increases in particle size contribute to better flowability and compaction, particularly in SD 4, which has demonstrated superior powder properties.

The SEM analysis thus complements the particle size data by visually confirming the relationship between the shape, size, and uniformity of the spray-dried particles. This further supports the conclusion that the spray-drying process was effective across different maltodextrin and gum arabic ratios, although there were variations in particle size and morphology among the samples.

The particle size of the elderberry powder reported by Gagneten et al. (2019) was 6.06 ± 0.59 μm, which is significantly larger compared to the particle sizes of the powders in this study [38]. The mean particle sizes of the present powders, ranging from 1.73 ± 1.27 μm to 2.20 ± 0.58 μm, are notably smaller. This difference suggests that the spray-drying technique and formulation parameters used in the present study produced finer particles. Smaller particle sizes are generally associated with enhanced dissolution rates and better homogeneity in formulations, which may offer advantages in applications requiring rapid solubility and uniformity, such as in pharmaceutical and nutraceutical products [39,40]. Furthermore, the particle morphology may play a key role in determining the shelf life of the final product, as its structure could influence both stability and degradation rates, essential factors for maintaining product quality during storage and distribution.

### 3.2. Encapsulation Efficacy of Encapsulated Elderberry Extracts Analyzed Using HPLC-DAD Analysis

**Figure 2:**
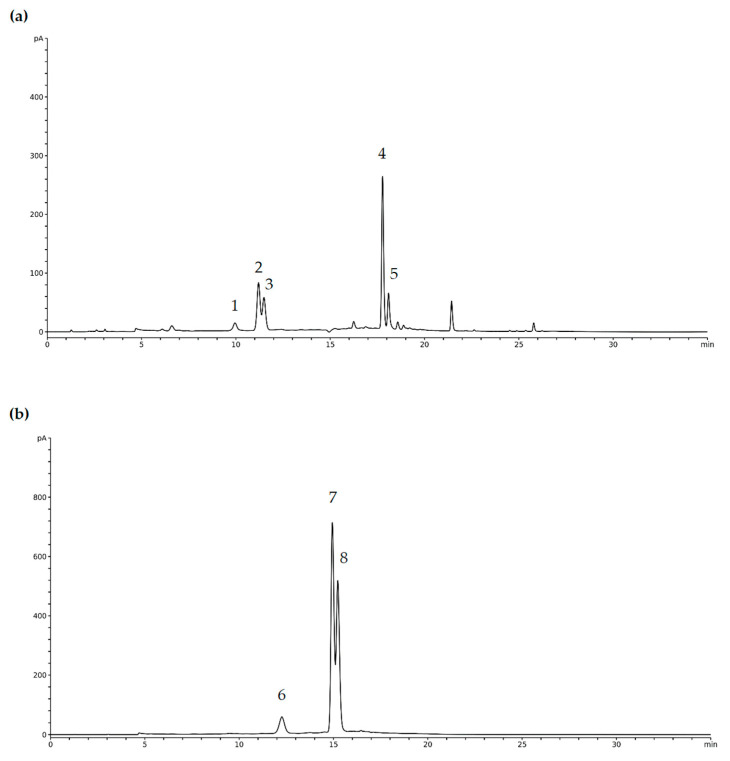
HPLC chromatograms of phenolic compounds from BRP extracts, detected at (**a**) 350 nm for 1—chlorogenic acid, 2—kaempferol derivate 1, 3—kaempferol derivate 2, 4—rutin, 5—isoquercitrin, and (**b**) 520 nm for 6—cyanidin 3-O-galactoside, 7—cyanidin 3-O-sambubioside, 8—cyanidin 3-O-glucoside.

The encapsulation of bioactive compounds has a crucial role in improving the functionality and stability of natural extracts, especially in the transition from liquid to powder forms [41]. This process is particularly important in the context of spray drying, a widely used technique that facilitates the preservation of bioactive components while providing a convenient format for various applications. In this analysis, the focus was on the identification and concentrations of bioactive compounds in the spray-dried powders SD 1–SD 6. The results are presented in Figure 2 and Table 3. By comparing these concentrations with the expected values in the pure extract with carriers (PE + C), the impact of the encapsulation process on the preservation of these valuable compounds can be better understood. The results will clarify how factors such as the inclusion of encapsulating materials and the spray-drying process influence the final concentration of bioactives, ultimately guiding improvements in formulation strategies aimed at optimizing both efficacy and stability.

Considering the comparison with the PE + C, the results indicate a moderate decrease in the concentrations of bioactive compounds across the spray-dried powders (SD 1 to SD 6). This decrease is expected due to the encapsulation process and the adding of the carriers maltodextrin and gum arabic.

For instance, the concentration of cyanidin 3-sambubioside in PE + C is 19.16 mg/g, while in SD 1, it is 17.55 mg/g, representing an encapsulation efficacy of 91.60%. Similarly, cyanidin 3-glucoside concentration decreases from 15.18 mg/g in PE + C to 14.20 mg/g in SD 1, with an encapsulation efficacy of 93.54%. The observed encapsulation efficacies across the SD samples are relatively high, demonstrating that the spray-drying process was effective in preserving a substantial portion of these bioactive compounds. This is especially important because cyanidin 3-sambubioside and cyanidin 3-glucoside are potent anthocyanins known for their strong antioxidant, anti-inflammatory, and antiviral properties, contributing to improved cardiovascular health and potential cancer prevention [42,43]. Ravichandran et al. (2023) showed that anthocyanin content in elderberry extracts ranged from 10.3 to 14.7 mg of cyanidin 3-glucoside equivalent per g of powder when encapsulated [44]. This variation is often influenced by the specific conditions of the spray-drying process and the carrier agents used. In the present study, this range of concentration was measured only for cyanidin 3-glucoside, which indicates that the powders in this research encapsulated a higher concentration of total anthocyanins.

The reductions in concentrations of other compounds, such as cyanidin 3-galactoside, rutin, and chlorogenic acid, across the SD samples are consistent with the expected dilution due to the presence of carriers. However, the encapsulation efficacies remain robust, particularly for compounds like kaempferol derivate 1 and kaempferol derivate 2, which show high efficacy in samples such as SD 5 (97.86% for kaempferol derivate 1) and SD 4 (98.57% for kaempferol derivate 2). Kaempferol derivatives are known for their strong antioxidant, anti-inflammatory, anticancer, and cardioprotective properties, making them valued compounds for promoting overall health and preventing chronic diseases [45,46].

In comparison to PE + C, the slight decreases in concentration observed in the SD samples underscore the impact of the encapsulation process. The results suggest that while there is some loss of bioactive compounds during spray drying, the overall encapsulation efficacy is sufficient to maintain the functional properties of the powders. This aligns with the study’s goal to optimize the spray-drying process for maximum encapsulation efficacy and stability of bioactive compounds in the final product.

Studies have demonstrated that employing gum arabic and maltodextrin as carriers can achieve high encapsulation efficiency rates, typically around 80–90% for phenolic compounds. The high encapsulation efficiency values are attributed to the ability of these carriers to form stable protective matrices around the bioactive compounds, which helps in preserving their antioxidant capacity and enhancing their release during digestion [47]. The encapsulation efficiency of cyanidin 3-glucoside in black elderberry pomace extracts encapsulated by spray drying with beta-glucan was reported to be approximately 84% [48]. These findings highlight the potential of spray-drying techniques to improve the stability and usability of elderberry pomace, offering a way to valorize this by-product in functional foods and nutraceuticals. In direct comparisons, gelatin and whey protein often fall short in terms of long-term stability, as they do not offer the same level of protection against moisture and oxidation. Therefore, gum arabic is favored in applications requiring enhanced stability of sensitive bioactives under challenging conditions [49].

Another encapsulation technique that is also frequently used, freeze drying, ensures excellent preservation of heat-sensitive bioactives and achieves an encapsulation efficiency of over 95% for anthocyanins. However, it leads to powders with poor flowability, higher hygroscopicity, and higher energy and time requirements [50]. In contrast, spray drying offers a more cost-effective and scalable alternative, where the encapsulation efficiency of phenolic compounds is between 80% and 95%, depending on the carrier composition and drying conditions. In our previous study, the PGSS technique, using supercritical CO₂, achieved around 50% encapsulation efficiency for rutin from the UAE extract, which is similar to the results obtained using spray drying of the same extract [11]. The advantage of the PGSS technique is its high efficiency for lipophilic compounds, while its limitation lies in the lower efficiency for hydrophilic compounds, in this case, anthocyanins, due to their low solubility in the supercritical medium. Spray drying is often preferred for large-scale production due to its cost-effectiveness and scalability, while PGSSs and lyophilization, although better at preserving bioactives, are more expensive and less suited for large-volume commercial applications due to the batch-processing nature of lyophilization and the high-pressure requirements of PGSSs [51].

## 4. Conclusions

This study demonstrates that maltodextrin and gum arabic are effective agents for encapsulating elderberry extract, with EYs ranging from 75.36% to 83.84%. The highest yield was achieved with SD 1 (100% maltodextrin), with an EY of 83.84%. This formulation exhibited superior matrix-forming ability, which is crucial for preserving bioactive compounds. However, while SD 1 achieved the highest EY, flow and compressibility analysis revealed it had the poorest flow properties (Carr index of 39.91%) and the highest compressibility. In comparison, SD 4 (40% maltodextrin, 60% gum arabic) showed significantly better flow (Carr index of 27.34%) and compressibility, making it more suitable for processing. Particle size analysis indicated average particle sizes ranging from 1.73 μm to 2.20 μm, with SD 2 exhibiting the most consistent particle size distribution. Regarding the encapsulation of bioactive compounds, SD 1 retained a concentration of cyanidin 3-O-sambubioside at 17.55 mg/g (91.60% efficiency) and cyanidin 3-O-glucoside at 14.20 mg/g (93.54% efficiency). However, SD 4 demonstrated high efficiencies for phenolic compounds, such as kaempferol derivate 1 (97.86% in SD 5) and kaempferol derivate 2 (98.57% in SD 4), highlighting its potential for preserving bioactivity.

Considering all these factors, although SD 1 has the highest EY, SD 4 stands out as the better option due to its balanced flow and compressibility properties, making it more suitable for practical applications. Further optimization of drying conditions and ingredient proportions is recommended to maximize the benefits of these encapsulation agents, particularly in preserving bioactive compounds. Potential scale-up considerations include the hygroscopic nature of gum arabic, which may require careful moisture control to maintain powder stability in large-scale production. Additionally, due to the relatively higher cost of gum arabic, it is essential to assess the economic feasibility of the process and the added value of the final product to ensure a balanced cost–benefit ratio. Additional research is needed to evaluate powder stability in storage, interactions with food matrices, and sensory attributes in applications. A thorough cost–benefit assessment is also essential to determine the economic feasibility of this encapsulation approach, particularly in the context of consumer acceptance, which depends on sensory properties, perceived naturalness, and potential health benefits. Furthermore, investigating the encapsulation efficiency of other bioactive compounds under similar conditions could further demonstrate the versatility of this technique and provide valuable insights into its broader applicability.

## Figures and Tables

**Table 1:** Nomenclature of powders formed using spray-drying process with carriers, maltodextrin and gum arabic.

Code	Maltodextrin (%)	Gum arabic (%)
SD 1	100 (1.45 g)	0 (0.00 g)
SD 2	80 (1.16 g)	20 (0.29 g)
SD 3	60 (0.87 g)	40 (0.58 g)
SD 4	40 (0.58 g)	60 (0.87 g)
SD 5	20 (0.29 g)	80 (1.16 g)
SD 6	0 (0.00 g)	100 (1.45 g)

**Table 2:** Physical characteristics and flowability of obtained black elderberry pomace microparticles.

	MC	EY	BD	TD	CI	HR	PS
SD 1	5.63 ± 0.15 ^a^	83.84 ± 3.39 ^a^	160.25 ± 5.07 ^c^	266.67 ± 3.79 ^ab^	39.91 ± 0.76 ^a^	1.66 ± 0.08 ^a^	1.73 ± 1.27 ^c^
SD 2	4.95 ± 0.20 ^b^	80.00 ± 3.70 ^a^	162.63 ± 3.59 ^bc^	253.12 ± 6.44 ^bc^	35.75 ± 0.90 ^b^	1.56 ± 0.06 ^ab^	1.94 ± 0.08 ^bc^
SD 3	4.20 ± 0.12 ^c^	77.44 ± 3.57 ^a^	194.36 ± 5.79 ^a^	277.14 ± 3.53 ^a^	29.87 ± 0.87 ^c^	1.43 ± 0.06 ^bc^	2.05 ± 0.10 ^ab^
SD 4	3.85 ± 0.10 ^c^	75.36 ± 3.54 ^a^	178.61 ± 7.99 ^ab^	247.22 ± 3.02 ^c^	27.75 ± 0.62 ^c^	1.38 ± 0.01 ^c^	2.20 ± 0.58 ^a^
SD 5	3.95 ± 0.18 ^c^	78.23 ± 2.87 ^a^	187.23 ± 8.21 ^a^	258.33 ± 12.34 ^bc^	27.52 ± 1.37 ^c^	1.38 ± 0.03 ^c^	2.03 ± 1.65 ^ab^
SD 6	4.10 ± 0.09 ^c^	81.25 ± 1.12 ^a^	185.96 ± 1.87 ^a^	255.55 ± 3.25 ^bc^	27.23 ± 1.11 ^c^	1.37 ± 0.02 ^c^	2.01 ± 1.02 ^ab^

Different letters within a column indicate a significant difference between samples at *p* < 0.05. Moisture content, MC; Encapsulation yield, EY; Bulk density, BD; Tapped density, TD; Carr index, CI; Hausner ration, HR; Particle size, PS.

**Table 3:** Concentrations of phenolic compounds in powders (µg/g powder) obtained by spray drying black elderberry pomace UAE extracts with maltodextrin and gum arabic.

Code	C3Sam ** (mg/g)	C3Glu ** (mg/g)	C3Gal ** (mg/g)	Rut ** (mg/g)	IQ ** (mg/g)	CA ** (mg/g)	KMP1 **/*** (mg/g)	KMP2 **/*** (mg/g)
PE	28.34 ± 0.43	22.77 ± 0.34	2.19 ± 0.04	8.86 ± 0.27	2.89 ± 0.06	2.10 ± 0.01	4.17 ± 0.06	0.66 ± 0.02
PE + C	19.16 ± 0.21 ^a^	15.18 ± 0.23 ^a^	1.46 ± 0.01 ^a^	5.91 ± 0.25 ^a^	1.94 ± 0.03 ^a^	1.40 ± 0.01 ^a^	2.78 ± 0.05 ^a^	0.44 ± 0.01 ^a^
SD 1	17.55 ± 0.35 ^b^ (91.60%) *	14.20 ± 0.18 ^ab^ (93.54%) *	0.87 ± 0.03 ^b^ (59.59%) *	3.17 ± 0.13 ^b^ (53.67%) *	1.67 ± 0.02 ^b^ (86.23%) *	1.10 ± 0.05 ^c^ (78.57%) *	0.44 ± 0.02 ^bc^ (15.83%) *	0.21 ± 0.01 ^d^ (47.73%) *
SD 2	15.94 ± 0.49 ^c^ (83.19%) *	12.73 ± 0.23 ^cd^ (83.86%) *	0.82 ± 0.03 ^bc^ (56.16%) *	3.26 ± 0.12 ^b^ (55.19%) *	1.65 ± 0.07 ^b^ (85.20%) *	1.18 ± 0.05 ^bc^ (84.29%) *	0.53 ± 0.01 ^b^ (19.06%) *	0.27 ± 0.01 ^b^ (61.36%) *
SD 3	14.36 ± 0.38 ^d^ (74.95%) *	11.49 ± 0.51 ^d^ (75.69%) *	0.74 ± 0.01 ^d^ (50.68%) *	2.82 ± 0.11 ^c^ (47.74%) *	1.54 ± 0.07 ^c^ (79.52%) *	1.30 ± 0.07 ^a^ (92.86%) *	0.38 ± 0.01 ^c^ (13.67%) *	0.18 ± 0.01 ^e^ (40.91%) *
SD 4	15.80 ± 0.50 ^cd^ (82.46%) *	12.70 ± 0.32 ^cd^ (83.66%) *	0.81 ± 0.02 ^bcd^ (55.48%) *	3.09 ± 0.08 ^bc^ (52.31%) *	1.63 ± 0.06 ^bc^ (84.16%) *	1.38 ± 0.06 ^a^ (98.57%) *	0.44 ± 0.01 ^bc^ (15.83%) *	0.22 ± 0.01 ^cd^ (50.00%) *
SD 5	16.32 ± 0.59 ^bc^ (85.18%) *	13.07 ± 0.63 ^bc^ (86.10%) *	0.84 ± 0.01 ^b^ (57.53%) *	3.19 ± 0.14 ^b^ (54.01%) *	1.66 ± 0.07 ^b^ (85.71%) *	1.37 ± 0.04 ^a^ (97.86%) *	0.47 ± 0.02 ^bc^ (16.91%) *	0.24 ± 0.01 ^c^ (54.54%) *
SD 6	15.10 ± 0.71 ^cd^ (78.81%) *	12.10 ± 0.55 ^cd^ (79.71%) *	0.75 ± 0.02 ^cd^ (51.37%) *	2.99 ± 0.07 ^bc^ (50.62%) *	1.61 ± 0.02 ^bc^ (83.13%) *	1.29 ± 0.04 ^ab^ (92.14%) *	0.41 ± 0.01 ^bc^ (14.75%) *	0.20 ± 0.01 ^de^ (45.45%) *

* Percentage of preserved active compounds in powders (encapsulation efficacy). PE—concentration in pure extract; PE + C—expected concentrations in obtained powders (mixture of pure extract and carrier); C3Sam—cyanidin 3-O-sambubioside; C3Glu—cyanidin 3-O-glucoside; C3Gal—cyanidin 3-O-galactoside; Rut—rutin; IQ—isoquercitrin; CA—chlorogenic acid; KMP1—kaempferol derivate 1; KMP2—kaempferol derivate 2. ** Letters that differ within a column represent a significant difference between samples at *p* < 0.05. *** Expressed as kaempferol.

## Data Availability

The original contributions presented in the study are included in the article, further inquiries can be directed to the corresponding author.
